# Online group projects in higher education: persistent challenges and implications for practice

**DOI:** 10.1007/s12528-023-09360-7

**Published:** 2023-03-24

**Authors:** Helen Donelan, Karen Kear

**Affiliations:** School of Computing and Communications, Faculty of Science, Technology, Engineering and Mathematics, The Open University, Walton Hall, Milton Keynes, MK7 6AA UK

**Keywords:** Systematic review, Online group projects, Collaboration, Team-work, Participation, Pedagogic design

## Abstract

With the rapid adoption of online learning across higher education, there is an urgent need to identify its challenges and ways of addressing them. Online group projects, in particular, present significant issues for educators. This paper presents the findings of a systematic literature review identifying the key challenges of online group projects, together with strategies to address them. From a corpus of 114 recent papers, the 57 most relevant were analysed, to identify themes related to challenges and strategies. Key challenges were: low and uneven participation by students; a lack of clarity and preparation for students; and poor relationships. Strategies for addressing challenges were: careful design of projects, particularly regarding fair assessment; clear guidance and preparation of students; and practical and emotional support throughout, to encourage confidence and engagement. The findings of this review will enable educators to design and facilitate online group projects which students find rewarding and valuable.

## Introduction and background

Group work, where students learn by collaborating and working with each other, is an integral, and sometimes compulsory, part of higher education qualifications. There are several reasons for this: collaborating with other students is a valuable way of learning (McConnell, 2005); and team-work is high on the employability skills agenda (Winterbotham et al., 2018). Employers often express their requirements in terms of skills rather than subject knowledge, and interpersonal and team-working skills rate highly. Employers want staff who can work well with others.

### Online group work

Until recently, most group work in higher education was conducted face-to-face. However, particularly since the 2020 pandemic, there has been a mass move towards online learning (Rapanta et al., 2020) where students learn through accessing teachers, peers and content via the internet. This often involves online group work, where group activities are negotiated and carried out using communication technologies. Such online groups may vary in size and focus, from small teams of students working together on a specific project to online classes taking part in free-flowing discussions.

Research focussing on students working together online in higher education has been plentiful (e.g. Garrison et al., 2000; Hiltz & Goldman, 2005; McConnell, 2006; Oliveira, 2011; Chang & Kang, 2016). It has provided valuable insight into the problems faced by students and educators. These problems include low participation by students and low levels of student satisfaction (Brindley et al., 2009; Kreijns et al., 2003). Some studies have offered solutions or recommendations (e.g. Roberts & McInnerney, 2007), typically intending to improve one or more of: student engagement; student perceptions or satisfaction; student performance or skills development. Now that adoption of online education is rapidly increasing, this body of research needs to be brought together systematically, in order to draw clear lessons for educators. Effective approaches to online group work are urgently needed, so that students can acquire skills online that were previously taught and developed on campus.

Online group projects are the focus of this paper – as these can provide authentic contexts where students are developing employability skills through working, learning and producing something together online. Students should learn to work as part of a virtual team, as this has become central to most industries and careers (Bakken, 2018). We define an online group project by the characteristics listed below. These characteristics are common to the contexts and activities described in many studies, for example Thomas and MacGregor (2005), Bergeron and Melrose (2006), An et al. (2008), Oliveira et al. (2011), and Donelan and Kear (2018):

- Students working in small groups, using online tools as the primary means of communication.
- Groups working to achieve a particular outcome, complete a defined task, or produce something.
- Groups having a defined membership, with all members expected to contribute to the outcome.

Employability skills are at the centre of why online group projects are important in higher education; employability has become a key concern for Higher Education Institutions, as part of a focus on graduate attributes (Wong et al., 2022). To fully develop workplace-relevant skills, it is particularly important that tasks set are authentic i.e. provide realistic contexts which align with workplace requirements. Herrington et al. (2010) say that this means learners should be required to collaborate, reflect, and develop their own approaches to carrying out complex tasks. In addition, both Herrington et al. (2010) and Lombardi (2007) highlight the need to ensure that tasks are based on real world tasks that mirror professional practice.

Another key consideration when designing online group projects relates to how students are expected to work together. According to the definition of online group projects above, students have a common goal and are all expected to contribute to the specific outcome or deliverable. But how students get to that outcome can vary greatly. True collaboration takes place when students’ individual tasks are mutually dependent, and therefore need to be constantly negotiated and shared (Oliver et al., 2007). Some group projects, however, are more suited to a cooperative style of working, which allows tasks and responsibilities to be divided between group members (Paulus, 2005). In this approach students work alongside each other, bringing their separate contributions together later in the project, rather than working together throughout. This may also, in some circumstances or professions, be a more authentic way of carrying out group projects. The different ways in which students work together in online group projects is considered during the analysis later in this paper.

This paper reports a systematic literature review to discover what progress has been made in addressing the challenges of online group projects in higher education. The studies reviewed tend to focus on the evaluation of a project in a specific context, often through soliciting the views of participants. The contexts of these studies differ regarding, for example:

- Cohort demographics (age, educational and occupational backgrounds)
- Collaboration tools used, and methods for working together
- Size and heterogeneity of groups
- Structure of the task set.

This variation in contexts needs to be taken into account when considering the challenges of online group projects, and strategies to address them; we have tried to do this when considering the various studies that we have reviewed. Nevertheless, the wide variety of contexts can make it difficult to draw out generic findings in relation to challenges, strategies and the relations among them.

Because of the proliferation and diversity of research studies in this area, practitioners can find it difficult to identify effective strategies for designing online group projects, and for supporting students through the experience. The purpose of our review is to assist practitioners with these tasks. Based on our review and findings, we have categorised the many challenges which may arise, and linked these challenges to strategies that can be considered in order to address them. This should be particularly useful to educators new to online collaborative learning.

Our dual concern with challenges and strategies is embedded in our research questions:

- What are the main challenges in online group projects?
- What strategies have been proposed for addressing these challenges?

### The community of Inquiry framework

One of the main theoretical frameworks that is useful in considering how to design and support online group work is the Community of Inquiry (Garrison et al., 2000); we have therefore used this framework to aid our analysis of challenges and strategies. There is a significant body of work, developed over many years, which uses the Community of Inquiry as the basis for analysis of online collaborative learning (see, for example Fe, 2010; Chandler, 2022; Yu & Li, 2022). The findings of our review, as presented in this paper, are discussed in Sect. "Analysis and discussion" with particular reference to elements of the framework.

As explained by Fiock (2020), the focus of the Community of Inquiry framework is developing a community of learners through consideration of three main elements: social presence, teaching presence and cognitive presence. *Social presence* relates to online students feeling that other group members are real people (Gunawardena & Zittle, 1997). It is conceptualised by Garrison et al. (2000) in three categories: emotional (affective) expression; open communication; and group cohesion. All of these are important for online group projects. *Teaching presence* refers to the design and facilitation of online activities for learning. It is conceptualised as the following three aspects: instructional design and organization; facilitating discourse; and direct instruction. Good design and organisation are key to online projects, and facilitating discourse is also important; depending on the project, direct instruction may be less relevant, as students investigate the project topic for themselves. *Cognitive presence* refers to students constructing meaning together online. It is conceptualised as four phases: a triggering event; exploration; integration; and resolution. All of these are relevant to progress through an online group project.

## Methodological approach

### Systematic reviews

‘Systematic review’ in this paper means ‘research approaches that are a form of secondary level analysis (secondary research) that brings together the findings of primary research to answer a research question’ (Newman & Gough, 2020). A systematic approach to reviewing is recommended because of its rigour and comprehensiveness. It provides a sound way to gather evidence, provide a broad analysis, and bring clarity to the challenges that students and educators face.

Our approach identifies the common challenges of online group projects and the lessons that educators have learned about how to address these challenges. We identify good practices and look at how they may apply generally. Through analysis of 57 research studies, we discuss the nuances of the issues identified, and we answer the two research questions.

Because systematic reviews need to be reproduceable and transparent (Gusenbauer & Haddaway, 2019), the search process is documented below, including the database and search terms used.

### Search strategy

The literature search was performed using the ERIC (Education Resources Information Center) database – the largest database in the world for indexed and full-text education literature and resources (https://eric.ed.gov/). The ERIC database contains more than 250 journals. It was cross-checked to ensure it included journals known to the authors from related projects; it was found to include all such journals.

In the initial search of journal article titles, the following search terms were used, combined as follows:

(‘collaborat*’ OR ‘team’ OR ‘group’) AND (‘online’) AND (‘project’ OR ‘work’)

(The term ‘collaborat*’ was used to ensure that references to, for example, ‘collaboration’ or ‘collaborative’ were picked up.)

The ERIC database was initially searched for matching papers published between 2000 and 2018 (the date was later extended, as described at the end of this section). This produced 73 results. However, some key papers that the authors were aware of were missing. Other search terms were therefore trialled, new searches performed and the results analysed. For example, the term ‘learning’ was considered as an alternative to ‘project’ OR ‘work’; however this introduced a very large number of papers that were irrelevant to a study focussing on online group projects. The following narrower terms were therefore added and comprised the final search terms.

(‘collaborat*’ OR ‘team’ OR ‘group’) AND (‘online’ OR ‘elearning’ OR ‘e-learning’) AND (‘project’ OR ‘work’ OR ‘activit*’).

(The term ‘activit*’ was used to ensure that papers using the terms, ‘activity’ or ‘activities’ were included.)

Using this expanded set of search terms, all 73 of the previously found papers were included, together with the known papers which had been missing before, and several more. This gave a total of 101 papers. Following stages I and II, which are described below, and which were lengthy, two catch-up searches were performed again, to include any new papers that had been published. These catch-up searches ensured that papers over a 20-year period from 2000 to the end of 2020 were included. An additional three papers were identified in the first catch-up run, and a further 10 in the second, bringing the total to 114.

### Stage I: abstract review

In the first stage of the research, each of the 114 abstracts was independently reviewed by both authors of this paper, and its relevance judged to be: high (score 1), mid (score 2), or low (score 3). To qualify as ‘high relevance’ (score 1), abstracts had to concern online group projects (as defined within this paper) and had to indicate that challenges with the projects, and/or strategies for designing and implementing projects, were discussed or evaluated. Mid-relevance abstracts (score 2) were less obviously relevant, sometimes because the focus appeared to be on collaborative learning more generally, rather than group projects; however, the contexts seemed sufficiently relevant for further consideration. Low relevance abstracts (score 3) were too far outside of the scope of this research to warrant further scrutiny.

The two numeric scores, one from each author, for each abstract were then added to obtain an overall score. *Note that, in this scoring system, a low numeric score indicates high relevance.* Those whose overall score was 2, 3 or 4 (meaning at least one researcher felt it was highly relevant, or both felt it was of mid relevance) were retained for full paper analysis. Where scores differed by 2 (one author scored the paper as 1 and the other as 3), the whole paper was read and discussed before a decision was made about its inclusion. The remainder, with scores of 5 or 6 (meaning that both researchers felt they were of low relevance, or one mid and the other low relevance) were excluded from the next stage of analysis.

### Stage II: full paper review and initial themes

A total of 61 papers were identified through the abstract review stage described above. However, the authors were unable to find copies of 4 of these, so in total 57 papers were subsequently read (by the first author of this paper). Particularly relevant papers from which key findings emerged were also read by the second author, in order to clarify and strengthen developing themes.

During this stage of the analysis, three summary documents were created. The first document was used to summarise each paper in terms of the aims of the study, context, the approaches to group work described, and any key challenges and strategies identified. The second summary document was used to collate and develop potential themes around challenges. These initial themes were documented using descriptive titles with supporting extracts or notes under each theme and reference to the papers in which these appeared. See Table 1 for an example.

**Table 1:** Example of a developing theme around challenges

Theme	Examples
Scheduling/time issues	Time delays–taking too long to make decisions (including procrastination) [86, 85, 37, 35]
	Tasks taking longer due to ‘group work’ element [85, 37, 9]
	Different time zones [59, 21]
	Team members contradicting schedules [31, 21, 9]
	Different work paces [21] etc.…….

The third summary document did the same for strategies rather than challenges. These three documents were created in parallel, with summary information from the first being used to inform the challenges and strategies themes being documented in the second and third. The initial groupings of examples of challenges and strategies were used to develop initial themes. These are presented briefly in this paper (see Tables 4 and 5), but full discussion is focussed on the refined themes that emerged in the final stage of analysis, described below.

### Stage III: final themes

As the review progressed it became clear that many of the initial themes were entangled or closely related and needed refining. Some initial themes, particularly with respect to the challenges, were therefore merged, either because there was significant overlap, or because there was a suitable over-arching theme (see Table 6 in Sect. "Results from Stage III (final themes) on Challenges"). On the other hand, particularly with respect to the strategies, some initial themes were too broad, so more detailed themes were created, for example, where strategies targeted different issues or were focussed on improving specific aspects (see Table 7 in Sect. "Results from Stage III (final themes) on Strategies").

Discussion in this paper is structured around these refined themes, and examples from the studies are used to illustrate them. It also became apparent that there were important relationships between some of the themes. For example, some challenges sometimes occurred as a consequence of others.

## Results

### Results from stage I (abstract review)

The 114 abstracts were reviewed and scored on relevance by both authors, as described in Sect. "Stage I: abstract review". The inter-rater reliability is reported in Table 2. This shows the number and proportion of abstracts (out of the total 114) where: the two authors gave the same relevance score (agree); the scores differed by 1 (partially agree); and the scores differed by 2 (disagree).

**Table 2:** Inter-rater reliability

Difference in scores	Level of agreement	Number of abstracts	Proportion of total (%)
0	Agree	74	64.9
1	partially agree	35	30.7
2	Disagree	5	4.4

The scores from both authors were added to give an overall score. (*Recall that, in this scoring system, a low numeric score indicates high relevance.)* Twenty-one abstracts received an overall score of 2, meaning that they were ranked as high relevance by both authors. A further 17 abstracts had an overall score of 3 (were ranked as high relevance by one author and mid relevance by the other) and a further 23 had an overall score of 4 (were ranked as mid-relevance by both authors, or high by one author and low by the other). The remaining 53 abstracts received an overall score of 5 or 6, meaning that they were ranked as low relevance by at least one author. These papers were insufficiently relevant to be analysed further. A summary of the scores allocated to papers is provided in Table 3.

**Table 3:** Scoring of abstracts

Number of abstracts	Total relevance score	Further analysis in Stage II	Proportion of total (%)
21	2	Yes	18.4
17	3	Yes	14.9
23	4	Yes	20.2
21	5	No	18.4
32	6	No	28.1

Further analysis of 57 of the top scoring 61 papers was performed in Stage II (4 of the original 61 could not be found). Details of the 61 papers are summarised in the table in the appendix. The table includes the title, first author, date of publication, and the relevance scores allocated in the abstract review process.

### Results from stage II (full paper review and initial themes)

Summary notes were made on each paper and initial emergent themes identified that were relevant to the two research questions:

- What are the main challenges in online group projects?
- What strategies have been proposed for addressing these challenges?

This resulted in 14 initial themes on challenges and 8 on strategies. These are given in Tables 4 and 5. The tables also show in which papers examples of these themes occurred (using the listing numbers from the table in the Appendix). In the next section these themes are further refined, in some cases merged into overarching themes or split into subthemes, and discussed in detail, with examples highlighted from the papers.

**Table 4:** Initial themes: challenges

Challenges
Lack of clarity – about the task [2, 21, 53, 59, 107]
Scheduling/time issues [3, 9, 21, 31, 35, 37, 50, 53, 59, 78, 84, 85, 86, 111]
Unequal division of tasks [21, 53, 61, 84, 86]
Lack of clear roles / leader [3, 21, 53, 59, 61, 76, 79, 94]
Working cooperatively rather than collaboratively [2, 15, 21, 40, 50, 53, 61, 78, 109]
Lack of / late participation by some members [2, 15, 21, 31, 35, 40, 53, 59, 76, 78, 84, 86, 94, 107]
Ineffective communication (technology and tools) [2, 21, 22, 29, 31, 34, 35, 37, 50, 53, 59, 61, 68, 85, 86]
Lack of skills–how to work as a group [21, 50, 59, 61, 63, 79, 84, 86]
Lack of clarity–role of the tutor [2, 37, 65, 94]
Negative feelings–towards group marks [3, 39, 76, 94]
Negative feelings–towards group work generally [21, 35, 37, 53, 61, 77, 78, 84]
Negative feelings–emotional (fear, anxiety) [50, 53, 61, 64, 76, 94]
Weak/poor group relationships [2, 6, 15, 21, 28, 35, 49, 50, 53, 59, 61, 83, 86, 94, 107]
Failure to achieve closure (extended unsettled feelings) [76] [94]

**Table 5:** Initial themes: strategies

Strategie*s*
Group project design [1, 2, 6, 9, 12, 15, 20, 39, 40, 49, 50, 53, 59, 65 76, 78, 85, 102]
Group organisation [1, 6, 12, 28, 40, 49, 50, 53, 59, 61, 76, 78, 85, 86, 105]
Group relationships [1, 3, 6, 15, 21, 22, 35, 49, 61, 66, 76, 77, 78, 83, 85]
Role of the tutor [1, 20, 21, 28, 29, 39, 50, 61, 65, 76, 78, 83, 94, 109]
Mentoring/coaching [35, 63, 64, 79, 85, 97]
Tools and technology [34, 40, 53, 61, 65, 68, 81, 86, 94, 95, 102, 107, 108, 109, 111]
Teaching and preparation [1, 21, 59, 61, 76, 84, 85, 97]
Sharing, reflection and closure [20, 76, 78, 79, 84, 94]

### Results from stage III (final themes) on challenges

In this section, the final themes related to challenges are discussed. Nine final themes on this topic (which addresses the first research question) were identified. Table 6 shows how these 9 final themes relate to the initial 13 themes on challenges. For example, all initial themes that centred on negative feelings, and the reasons for them, were combined under one final theme. The final themes (labelled C1 to C9) are then discussed.

**Table 6:** Initial and final themes on challenges

Initial themes	Final themes
Lack of clarity–about the task Lack of clarity–role of the tutor	Theme C1: Lack of clarity
Scheduling/time issues	Theme C2: Scheduling or time issues
Unequal division of tasks Lack of clear roles / leader	Theme C3: Unequal division of tasks and roles
Lack of / late participation by some members	Theme C4: Late or lack of participation
Ineffective communication (technology and tools)	Theme C5: Ineffective technology and tools
Lack of skills–how to work as a group	Theme C6: Lack of preparation in group working skills
Negative feelings–towards (group) marks	Theme C7: Negative feelings
Negative feelings–towards group work generally	
Negative feelings–emotional (fear, anxiety)	
Weak/poor group relationships	Theme C8: Weak or poor group relationships
Failure to achieve closure (extended unsettled feelings)	Theme C9: Failure to achieve closure

#### Theme C1: lack of clarity

Examples identified here mostly refer to unclear guidelines for students about: the task and how to carry it out; what role the tutor will take during the group work.

In [59] ‘unclear instructional guidelines’ was identified as an impediment to online group work, and [21] cites ‘unclear objectives’ as one of the primary reasons that project teams fail. A lack of clear expectations about how students should divide the work or allocate roles also causes students problems, as does lack of transparency about how work will be graded [53], especially where group marks (where all students receive the same mark) are used [2]. Lack of explicit advice and guidance can result in other problems, such as: timing issues, due to more time needing to be spent on decision-making (theme C2); how to operate as a group and allocate roles and tasks (themes C2 and C3) [53]; and emotional problems such as worry (theme C7).

Lack of clarity about the role of the tutor (or facilitator) causes problems for students and for tutors. Students are uncertain under what circumstances they can contact a tutor if issues emerge and how this could affect their final marks [94]. For tutors it is difficult to decide when to intervene with individuals or groups [65].

#### Theme C2: scheduling or time issues

Scheduling or timing issues are a well-documented problem in online group work, with tasks often taking longer than expected [9, 37, 85], and procrastination can be a problem [35, 37, 85, 86].

Although face-to-face group work is also prone to these issues, online groups generally find it harder to resolve logistical issues such as scheduling [84, 107]. Many factors are at play here, depending on the context. Group members may be in different time zones [21, 53, 59, 111] or obliged to work to different schedules [9, 21, 31]. In distance learning contexts, students are often in paid employment or have caring responsibilities. Sometimes students simply work at different rates [21].

Some scheduling issues occur because groups do not establish a proper work plan and are therefore constantly reacting to deadlines [50]. This can be symptomatic of unclear instructions (theme C1) or students’ lack of preparation before the work starts (theme C6), which means they do not develop the necessary skills to schedule activities or account for unanticipated lack of participation by some group members (theme C4). Another factor that can affect timing is the communication technology used; this is discussed in more detail later (theme C5).

#### Theme C3: unequal division of tasks and roles

Examples here are often due to students’ perceptions of how work is distributed within a group, including the role of leader. A perceived unequal division of the workload often causes bad feelings or negative perceptions of group work (see more in theme C7 later).

In [86], effective groups had a perceived leader and felt that work was evenly distributed, with some members taking on more small tasks and others fewer large tasks. In contrast, low performing groups were characterised by a lack of leadership. Where there was a (self-perceived) leader in these groups, this person felt that tasks were unfairly distributed, and that he or she bore most responsibility.

In [2], tutors who were responsible for supporting students and assessing the work felt, even more strongly than students, that tasks were unevenly distributed. Tutors felt that the more technically competent students did most of the work. In [61] three of the four groups had an inequitable distribution of work because of some members falling short, leaving others to ‘pick up the slack’. This provides a link later with theme C4 (Late or lack of participation).

Generally, a lack of clear roles for group members and a failure to agree a process for making decisions (either via a leader or otherwise) have a detrimental effect on group performance and cohesion [21, 53, 59, 61, 105]. As with the previous theme, this can result either from lack of guidance (theme C1) or from lack of skills in how to work as a group (theme C6). Having self-appointed leaders does not always succeed and is dependent on the group dynamics. For example, in some cases other students see leadership as dictatorship [59, 86]. Many students, not wishing to assume a leadership role, are reluctant to initiate communications and are glad when someone else assumes leadership [53]. In [3] several works are cited claiming that the more conscientious students assume the leadership role.

#### Theme C4: late or lack of participation

This is one of the most commonly cited difficulties in online group work; accountability is difficult to achieve. Group members often do not know each other; and communication technologies make it easier for students to ‘lurk’ or engage very little.

Absent or non-participating group members (either from the start of a project or disappearing part way through) are mentioned in many studies [2, 31, 35, 40, 59, 76, 84, 86, 94, 107]. This issue can contribute towards low performance in groups [31], unequal division of tasks [61] (theme C3), poor relationships (theme C8) and negative feelings (theme C7) [78, 84].

In [78] the authors provide a summary of the ways non-participating students have been referred to in earlier studies: ‘slackers’ (Payne & Monk-Turner, 2006), ‘social loafers’ (Shiue et al., 2010), and ‘free riders’ (Roberts & McInnerney, 2007). These terms imply’irresponsible students’ [15] who become a burden, doing less than everyone else, but potentially still getting the same marks. However [21], citing Roberts and McInnerney (2007), gives another reason for students withdrawing: they may feel ignored or that their contributions are not valued. Alternatively, students may not feel confident speaking up, due to dominant members or possible conflict [2]. Non-participation does not always connote irresponsibility.

#### Theme C5: Ineffective technology and tools

Ineffective communication tools, or the ineffective use of tools, is a common problem in online group work. There are several dimensions to the examples collated from the literature.

Technical problems, for example problems downloading other group members’ work, were identified in [31, 35, 59]. Students may lack ability or confidence with the tools provided and can lose the opportunity to develop technical skills if more confident students take over [15]. Providing tools with limited functionality, to create a simpler experience, may have an adverse effect on more capable students, resulting in feelings of frustration [2] (theme C7).

The types of communication supported by the tools also need to be considered. Online forums allow students to participate in discussions irrespective of time and availability, whilst providing a permanent record of interactions that students and tutors can use to keep track of group progress. However, forums can introduce a significant lag between posts [78], with the potential to cause delay to the work (theme C2). Keeping track of messages can also be time-consuming and difficult [37, 61, 85, 86].

With synchronous approaches, students’ conflicting schedules may lead to the exclusion of some group members [53]. Problems associated with relying solely on text-based modes of communication have been highlighted in some studies [29, 59]; for example, misunderstandings due to the lack of social or emotional cues, and feelings of isolation.

#### Theme C6: lack of preparation in group working skills

Several of the problems already discussed (see themes C2 and C3) can occur when students are inadequately prepared in the skills needed for group work.

In [21], a lack of essential group work skills is identified as a main problem. Symptoms of a lack of preparation in these skills were: absence of clearly defined roles, inadequate planning and scheduling, and an inability to deal with absent members or conflicts.

Several studies highlight particularly important attitudes and skills; if individual students lack these, it can impair group work. For example: a sense of individual accountability and consensus building [59]; giving and taking criticism [61, 63]; defining goals and constructively commenting on decisions [50]; conflict resolution and management, and negotiation [83, 76, 78] are all seen as key to successful group work.

#### Theme C7: negative feelings

Although some students realize the benefits of group work [35], negative feelings are extremely common.

Feelings can range from apathy, which seems linked with poor motivation and attitude [21, 53, 61], to outright hostility (Roberts & McInnerney, 2007), especially if students are inadequately prepared (theme C6). Whereas some online students appreciate the opportunity to work collaboratively, others feel it contravenes their decision to study online as it reduces their flexibility about when to study [37]. The use of group marks is a particular cause for grievance, especially when based on prior experiences of shared group marks that students felt were unfair [39, 76, 94]. Studies mention negative emotions such as fear [61], anxiety [2, 50, 76, 94], frustration [61, 64, 94] and stress [53]. These can be caused by: students being left to self-select groups [76]; conflict [94] or dominant personalities [2] (see theme C8 below); a lack of trust between group members [50]; and irresponsible group members [53].

#### Theme C8: weak or poor group relationships

Weak or poor relationships between group members can inhibit successful group work, and lead to negative perceptions (theme C7). Many of the papers reviewed cite relationship issues, both to identify them as a problem and to suggest reasons for them.

Weak relationships can be caused by students having insufficient time or opportunity for relationship building. This can be a problem with the design of the project or preparation of students (themes C1 or C6), as group formation (Tuckman, 1965) may take more time online. A lack of social presence [35, 36, 86, 97] and difficulties in building trust online [61] can affect group relationships. A group that does not build strong relationships, or a cohesive teamwork ethic, from the beginning can suffer from other challenges as a result.

Poor relationships can also emerge, or initially good relationships deteriorate, if other problems occur, such as personality clashes and disagreements [2, 86, 94]. This may happen at particularly challenging stages of the project. [83] identified that conflicts tend to arise three quarters of the way through a project, where results are expected but may not yet be delivered. Strong relationships help in resolving conflicts but weak relationships, leading to unresolved conflicts, can mean that members disengage from the project [83].

#### Theme C9: failure to achieve closure

Finally, as group work comes to an end, in what Tuckman and Jensen (1977) refer to as the ‘adjourning’ stage, challenges or problems may remain unaddressed and can continue to affect students after the group work has finished. This can produce extended unsettled or negative feelings [76, 94] (theme C7), and can lead to knock-on effects the next time students encounter group work, whether in an academic setting or otherwise.

### Results from stage III (final themes) on strategies

In this section, the final themes related to strategies are discussed. Ten final themes on this topic (which addresses the second research question) were identified. Table 7 shows how these 10 final themes (labelled S1 to S10) relate to the initial 8 themes on strategies. The final themes are then discussed individually.

**Table 7:** Initial and final themes on strategies

Initial themes	Final themes
Group project design	Theme S1: Preparing students and clarifying tasks
Teaching and preparation	Theme S2: Designing group projects for increased student motivation
	Theme S3: Designing group projects for cooperative or collaborative work
	Theme S4: Designing assessment to encourage participation
Tools and technology	Theme S5: Selecting tools and technology
Group organisation Role of the tutor	Theme S6: Careful group formation
	Theme S7: Advising on group organisation and roles
Group relationships	Theme S8: Managing and supporting group relationships
Mentoring /coaching	Theme S9: Mentoring and peer support
Sharing, reflection and closure	Theme S10: Sharing, reflection and closure

It became apparent during Stage II that two of the initial themes (Group project design; and Teaching and preparation) encompassed several more focussed themes. This resulted in these two themes being split into four (themes S1 to S4). In addition, elements from two other initial themes: (Group organisation; and Role of the tutor) were closely entangled; these were reorganised and separated out into two new themes (themes S6 and S7). Other initial themes were refined as evidence was gathered from the papers.

#### Theme S1: preparing students and clarifying tasks

A fundamental strategy for all educators designing and facilitating online group projects should be to prepare students, as a lack of preparation was found to be a cause of many of the challenges identified earlier. This includes being transparent about what is expected of students, such as giving examples of levels of contribution that are acceptable [76], and how they will achieve their goals. Some of the advice on how to provide clear guidance and prepare students prior to embarking on group work (thereby addressing themes C1 and C6) is summarised below.

The first step is convincing students of the value of group work [84]. Guidance material should be clear about the skills being developed and why they matter. Several studies emphasize the importance of guidance on all aspects of group work. In [65] the authors say this should include: the goal(s) of the project; essential tasks; participant roles; realistic timescales; keeping written records of meetings; and problem resolution procedures. Purpose, time frames, technology and assessment are mentioned in [85], and [84] emphasizes the need for succinct instructions on how to operate in an online environment.

In some approaches reviewed in this study, students are explicitly taught about the group work process, models for group work, and the roles that members can play—including the pros and cons of having a leader [1, 21, 59, 61, 76, 97, 105]. Negative feelings around group work (theme C7) could be improved through guidance materials clarifying supportive behaviours (e.g. showing empathy, clarification) and non-supportive behaviours (e.g. monopolizing discussions, defensive responses) [61]. In [21] training in team building and cohesion skills is specifically mentioned, and some studies discuss the importance of preparing groups for conflict resolution [21, 76].

Activities can be designed to prepare students and act as ice breakers – including opportunities for introductions, self-presentation and getting to know others [21, 85, 107, 108, 111]. Activities can include the development of ground rules [59, 61]. [76] advocates getting students to share strategies that they found helpful or unhelpful in previous group work.

The role of the tutor should be clearly stated for the benefit of both students and tutors. Tutor involvement may change during the project (see themes S6 and S7) but students should know: how to contact the tutor (both individually and as a group); whether the tutor will be observing interactions; and when students should ask for intervention and the process for initiating this [50, 61, 76, 78, 94].

#### Theme S2: designing group projects for increased student motivation

Motivating students to engage with group work can be difficult. Explaining the value of group work (as mentioned in theme S1) is one approach [84], highlighting the importance of group work skills for future careers, and for life more generally [35]. Studies found that a key to motivating students is ensuring tasks are based on authentic real-world problems, mirroring what professionals do, and building skills relevant to the workplace [2, 9, 20, 78, 113].

In [2] and [107] a motivating factor, and a way of promoting positive feelings (addressing theme C7), was enabling students to develop a product (e.g. a website) that they could be proud of. If students could show their final product to other groups, they were motivated to produce something very good [2]. Awarding marks or credit for individual contributions is also suggested for increasing student motivation [76, 85]. (This is discussed in more detail under theme S4.)

#### Theme S3: designing group projects for cooperative or collaborative work

It is important to consider how students are expected to work together. Should they work individually on subtasks, and then combine their work at the end, i.e. work cooperatively? Or should they work together on a product through constant interactions, i.e. work collaboratively? Although groups working cooperatively can function extremely effectively, collaboration skills are often what employers want and what designers of group projects wish to develop [15, 61, 78].

In [40], two online projects, each with several groups, were investigated. Despite differences in design of the two projects, the conclusion was that ‘if a group could find a way to split work, that is what it did’. Students like cooperative working as they are less dependent on others [109]; it potentially makes mark allocation less contentious, through individual marks, which may motivate students [53] (see theme S4 next).

In [15] a balance of collaborative and cooperative tasks is suggested, with collaborative tasks to encourage dialogue and group knowledge construction, and cooperative tasks allowing students to do some of the work separately. In [40] advice is given for designing group projects that are collaborative rather than cooperative. This includes creating ‘positive interdependence’ between tasks, to ensure each group member’s efforts are required to complete the overall tasks, and providing clear instructions on what collaboration should look like.

There are claims that cooperative working means less creativity and cohesion [15] and that ‘true training in group work’ comes from collaboration (Paulus, 2005 in [21]). However, cooperative working may alleviate difficulties such as unequal division of work (addressing theme C3) and time delays (addressing theme C2) caused by waiting for others to complete interdependent tasks. There is a balance to consider. Creating tasks which require positive interdependence, where students can only succeed if the group succeeds, encourages collaboration [59]. However, this may disaffect students and make them sceptical of group work (exacerbating theme C7).

#### Theme S4: designing assessment to encourage participation

One approach to assessing group work is to use group marks, where each member receives the same mark regardless of how much they contributed. This has already been shown to make many students sceptical about group work (theme C7). As discussed under theme C4, late or lack of participation by some group members is a primary challenge for groups. A way to increase participation is to foster individual accountability [59], most obviously through assessment and assigning marks.

Individualising marks recognises individual contributions [76, 113] and highlights uneven participation [65], especially if based on each member’s commitment, responsibility, and activity. Peer evaluation is one method that can be adopted, where group members rate each other, based on commitment to the project [53, 59]. However, care is needed with this approach as it may not always be done fairly and accurately [78]. Combining tutor, peer and possibly self-assessment can also be considered [50, 59]. Although individualising marks may be more burdensome for the tutor, some studies have shown that tutors nevertheless strive for fairness and the ‘best’ approach rather than taking the easiest [2, 39].

#### Theme S5: selecting tools and technology

The right selection of online tools for enabling online group projects is key; ineffective tools were shown to cause scheduling issues (theme C2) and negative feelings (theme C7). Tools should be easy to use, appropriate for the tasks, and should facilitate collaboration [53]; furthermore, a good combination of tools can encourage variation in the types of interaction [34, 86, 109]. Learning to use new tools was reported as motivational for some students [2, 34].

Much discussion has centred on the relative merits of synchronous and asynchronous technologies. In terms of synchronous technologies, Instant Messaging (IM) is most noticeably used and evaluated in the papers reviewed. In [95] it was found useful for task support and information exchange, and in [68] for coordinating joint decisions, and for making links between academic discussion, knowledge construction and task coordination. Opinions differ on whether IM is a good tool for social interactions and support. Task support rather than social support was observed in [95], but groups engaging in conversation via IM had a higher level of participation. In [68] social interactions using IM were observed. In general IM was found to be useful for moving things along [50, 68] although this depended on the context and whether different time zones were involved.

For asynchronous communication, forums were the most popular choice. Although forums usefully make participation visible [65], it was pointed out in [94] that forums hosted on a virtual learning environment and visible to tutors can make students wary about what they say. They do not want to appear less knowledgeable than their peers – in case they receive a lesser mark. For effective interaction, [61] suggests encouraging communication outside of the institution’s learning environment; [108] and [111] mention that students used social media.

Whereas earlier studies focus on forums, from around 2013 more mention is made of Web 2.0 tools such as wikis, blogs, website development tools [2, 27, 34], and collaborative spaces such as Google Docs [40, 53, 107]. These are designed for shared authoring, and can avoid duplication of work or keeping track of separate files [53]. In the context of asynchronous tools use, mobile phones and applications were suggested for reducing delays in responding [53] although surprisingly little is said in any of the papers about using mobile devices to facilitate online group work.

Although a few relatively recent papers mention the use of video or voice communications [e.g. 108, 109, 111], most of the methods of communication and tools discussed are text-based. Guidance (theme S1) is needed to prepare students for communicating in this way: for example, using an informal writing style, using appropriate abbreviations and expressing thoughts concisely [35].

#### Theme S6: careful group formation

This theme concerns how students are allocated to groups. What size groups should be used? Should group members have similar or mixed abilities? Are there cultural aspects to consider or different times that could cause scheduling problems (theme C2)? Is the tutor responsible for forming groups? These are all elements that can affect how well a group connects and performs.

Generally, studies agree that groups of fewer than five work best and are easier to manage [6, 12, 29, 53, 85]. However, if groups are too small, drop out or failure of a member to participate can have an impact on the rest of the group. Therefore, sometimes groups are chosen to have between five and eight students [2, 12]. Students generally feel more comfortable in smaller groups where they can get to know each other, feel they are better understood, and see their individual contributions recognised [6]. This helps promote more positive feelings and better group relationships (addressing themes C7 and C8). In [12] smaller groups performed better on convergent tasks (tasks that ‘converge towards a single goal’), whereas larger groups performed better on divergent tasks (where more than one outcome is possible and involving greater discussion).

Where composition of groups is discussed, diverse groups seem to be favoured. In [6] students preferred diverse groups because of the multiple views and insights. In [86] it is suggested that personality type scales can be used to form heterogenous groups. Avoiding minorities in a group is suggested in [77], as having minorities can promote negative feelings (theme C7); and [21] suggests that a distribution of skills, methods and work ethics within a group can promote interaction and broader investigations. In [28] it was found that in international group projects, students from the same culture tended to interact more with each other and that cross cultural or international collaboration needs more facilitation – so the role of the tutor may be more significant [111].

Letting students form their own groups can be stressful for them [76], so ideally the tutor should form the groups. This is not always easy in online settings if tutors do not know the students, their abilities, or levels of commitment. [76] found that students greatly appreciated ‘thoughtful composition’ (p.73) of groups. As discussed in theme S1, the design and scheduling of a group project should allow tutors to get to know students before allocating them to groups, and allow students within a group to get to know each other (addressing theme C8).

#### Theme S7: advising on group organisation and roles

Following formation, groups proceed to organise themselves, which is the focus of this theme. The early stages of a group project, the ‘forming’ stage (Tuckman, 1965) where students are getting to know each other, can involve allocating roles and tasks to group members. The importance of clearly explaining group roles, and how to operate as a group, prior to starting have already been highlighted in theme S1; there are ways of continuing to reinforce these concepts. Creating activities that help students understand and assign group roles is useful [49], to avoid problems related to division of tasks and roles (theme C3). The tutor’s role can include articulating expectations of students [76, 94], initiating networking opportunities and posting introductions [94] to help groups form trust and respect [1] and build stronger group relationships (addressing theme C8).

Creating activities that enable groups to establish a set of ‘ground rules’ or a ‘team agreement’ is a recommended approach [6, 15, 61]. Periodically reviewing these rules or agreements is important, as they can easily be forgotten as group work intensifies [61]. In terms of roles, whilst all groups are different, a common characteristic of successful groups is having a leader – particularly if this role can be agreed by the whole group and possibly distributed between several members [53, 105].

Finally, we return to the role of the tutor during the group work. Opinions differ on the level of tutor involvement once group work is underway. Although early involvement in the construction of groups and in support and facilitation [50] helps build trust [1], subsequently the tutor needs to step back to some degree. Their role becomes one described in [50] as ‘critical observer’. Tutors then need to: ‘monitor groups’ progress’ [78, 75]; ‘provide feedback on level of interactivity’ [83]; ‘ensure teams are functioning effectively’ [21]; and ‘give encouragement, making it clear when a student’s contribution was particularly astute or helpful’ [20].

In [76] and [109], students were reassured to know their tutors were present which may help alleviate negative feelings building up (theme C8). This is similar to findings in [94], where students wanted to know that tutors could give individual support and were available if needed; private email exchanges between student and tutor were suggested as a way of reassuring students. Several studies state that tutors should intervene where problems arise, such as by: prompting non-participating members [61, 76] or those participating insufficiently [78]; generally supporting groups that have difficulties [50, 21]; and pointing out when ground rules are not being observed [61].

#### Theme S8: managing and supporting group relationships

The two preceding themes focussed on the practicalities of forming and organising groups. Another key consideration, as we have seen in the challenges, is the emotional side of group work. Practicalities and emotional aspects cannot be completely separated, as effective group work often stems from good relationships between group members. Conversely, groups can break down because of poor or weak relationships (theme C8).

Establishing trust within groups is key [61, 76, 77]. Posting introductions and biographies helps, so it is worthwhile providing opportunities and activities for this [61]. The development of identity within a group, and self-representation, can create belonging, and help students to develop supportive relationships [1, 66]. As pointed out in [77], there is rarely time to develop deep interpersonal relationships. However, students do not always need deep relationships to build the necessary trust.

Reliability, dependability, and work ethic have been cited as the most important group member.qualities [1, 49]. If members demonstrate these traits early, and show they care about the task in hand, it can go a long way to help students feel connected to, and safe within, a group [77].

Fear, stress and worry are common negative emotions associated with group work. Preparing students and removing unnecessary uncertainties helps counteract these, as does ensuring that interventions – such as tutor involvement – can happen if needed [61] (theme C8). Conflict within groups is sometimes unavoidable. As shown in the previous section, personality clashes and disagreements can jeopardise group relationships and performance. But ensuring that students learn strategies for managing conflicts can help – and in some cases make a group stronger once it has emerged from a dispute [83]. In [78] is it suggested that absent members could be dealt with through conflict management within a group, instead of students taking on more work to make up for absentees, with issues being escalated to the tutor as a last resort (addressing theme C8).

#### Theme S9: mentoring and peer support

Besides tutor support, other strategies for supporting students were raised by some studies. Providing a dedicated forum was suggested in [35], and described in other studies, as a way to offer support via tutors and other students. Although a forum cannot replace the one-to-one support of a tutor, peer support can provide advice from a different perspective. Other studies suggest that online group projects allow students to develop skills as online leaders or forum moderators [97]; providing training and practice in these skills can be part of a group project [85].

Two studies focus on the use of ‘between-group collaboration’ and the mentoring of less effective groups by more effective ones [63, 64]. The studies found that between-group mentoring and reviewing was perceived favourably by students, and the performance of less effective groups improved through learning from others’ strengths and weaknesses. Enabling less effective groups to see other groups’ strategies made the projects more authentic, and motivated students to work harder and produce better work.

#### Theme S10: sharing, reflection and closure

Strategies identified here are concerned with how group projects are ended. As discussed in theme C9, if problems are left unaddressed, negative feelings can persist [76, 94] (theme C7) and affect students’ approaches to online group work in the future.

Students need opportunities to debrief and attain closure [94] and should be encouraged to reflect on what went well or badly [79], (addressing theme C9). Sharing with others what has been learned is considered helpful [94]. Sharing final products, where appropriate, has also been identified as a positive post-project activity. It celebrates groups’ achievements and encourages pride in their work [2, 94]. Including elements of self-reflection in a project can help students to evaluate the benefits and challenges of the experience, understand their own strengths and identify areas to improve [78].

## Analysis and discussion

In this section we discuss the themes related to challenges and strategies, together with the relationships among them. We return to relevant literature identified earlier in the paper, in particular to the Community of Inquiry model (Garrison et al., 2000), as many of the elements of this model align well with the findings of our review.

### Challenges

**Fig. 1:**
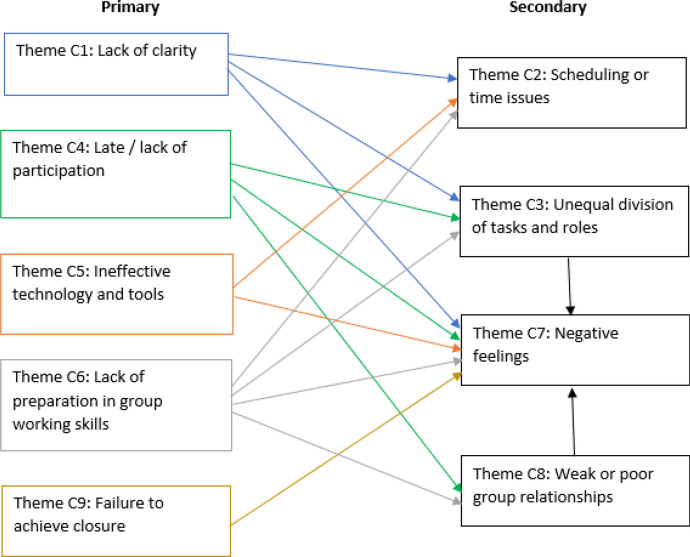
A summary of themes on challenges and the relationships between them

Our detailed analysis of papers relevant to our research questions revealed nine themes relating to the challenges of online group projects. By further consideration of the detailed findings presented in Sect. "Results from Stage III (final themes) on Challenges", including the links presented there between the various challenge themes, we identified some possible causative relationships between the challenges. This revealed that five challenges can tentatively be described as ‘primary’ because examples were found where they can lead to, or at least exacerbate, the other four. The primary challenge themes are: lack of clarity (C1); lack of participation (C4); ineffective technologies (C5); lack of preparation (C6); and failure to achieve closure (C9) (see Fig. 1). These primary challenges can contribute to secondary challenges: time issues (C2); unequal division of tasks (C3); negative feelings (C7); and poor relationships (C8). Figure 1 shows the various potential relationships among the challenge themes.

We return to the Community of Inquiry framework (Garrison et al., 2000) to consider, first, the primary challenges and why they so frequently occur. The ‘instructional design and organization’, aspect of *teaching presence* is particularly relevant. This is because the primary challenges arise if there is not a clear enough structure and narrative for students about what the tasks and goals are, and how students should work to achieve these goals. Lack of a clear design for group projects may result in some students failing to engage at all, and others struggling their way through.

The secondary challenges are more associated with the *social presence* aspect of the Community of Inquiry model: emotional (affective) expression; open communication; and group cohesion. In fact, a lack of social presence was identified in several studies to be the cause of poor group relationships (C8) and non-participation (C4); it results in students ‘holding back’, in order to avoid misunderstandings or conflict. Other examples were highlighted from the corpus of papers that showed how negative feelings were often associated with poor relationships and the breakdown of cohesive group working.

### Strategies to address the challenges

Regarding strategies to address the above challenges, two main types of theme emerged: strategies related to designing group projects and preparing students; and strategies related to group relationships and support. The first of these more directly address the primary challenges, whilst the second type more directly address the secondary challenges.

**Fig. 2:**
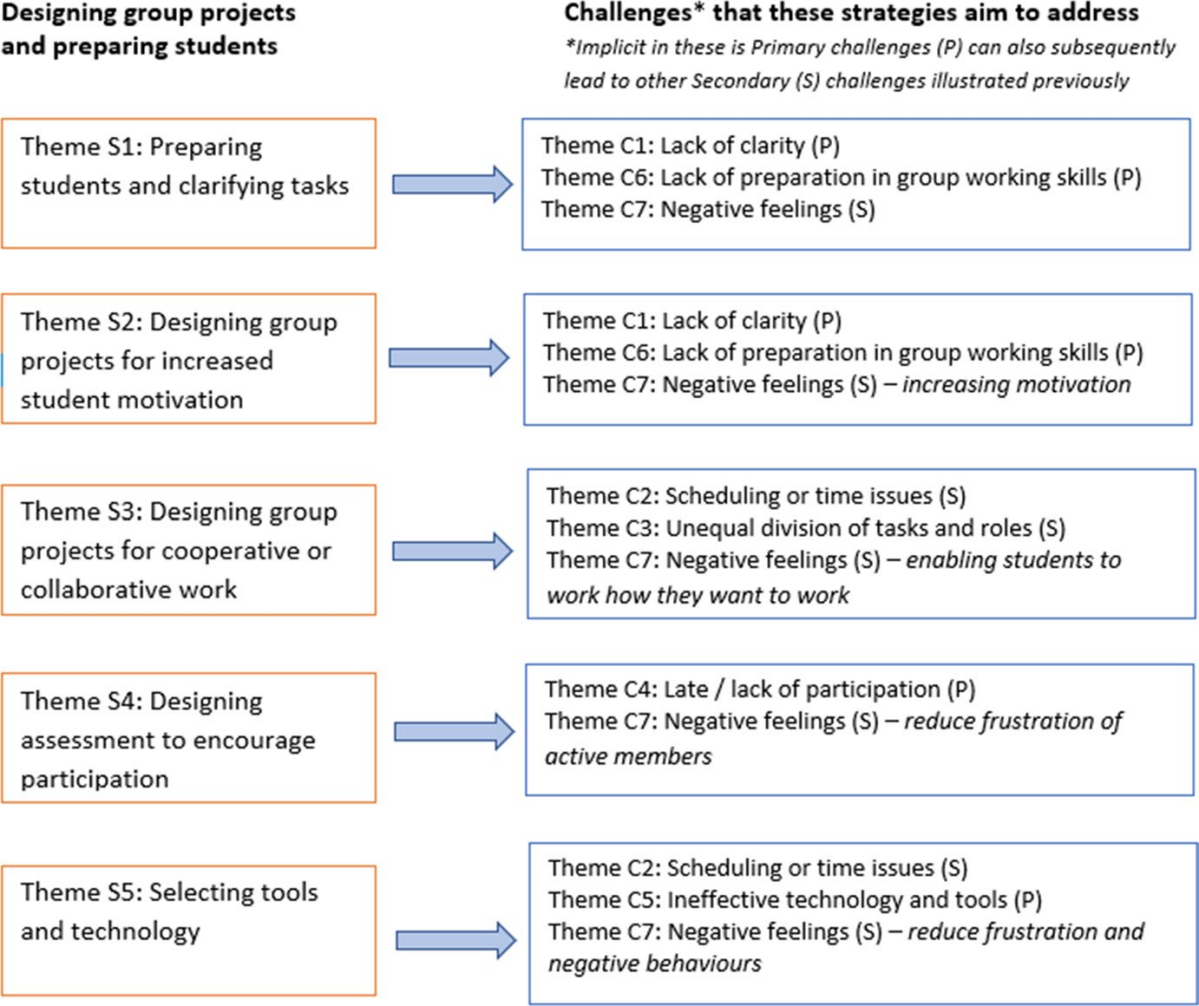
Strategies related to practical choices and preparing students

Firstly, we consider ‘Designing group projects and preparing students’ (see Fig. 2). These strategies are focussed on practicalities and educators’ initial design choices prior to students starting a group project–what students will be required to do, how they will do it, how the project will be assessed, and the guidance students will be given. These aspects directly influence how well-prepared students are as they embark on a project. Strategies in this first category were about: preparing students (S1), designing for motivation (S2), cooperative/collaborative work (S3), assessment for participation (S4), and selecting technologies (S5). As shown in Fig. 2, many of these strategies address the primary challenges identified by this review (and therefore also the secondary challenges).

All of these strategies relate to the ‘instructional design and organization’ element of *teaching presence* within the Community of Inquiry framework (Garrison et al., 2000). It is important to design online group projects so that tasks are clear, and students are well prepared. As summarised by Fiock (2020), activities should be well organised, easy for students to navigate, and reviewed for clarity and consistency. Authentic tasks, that match what professionals do in practice (Herrington et al, 2010; Lombardi, 2007), have been shown in this review to motivate students. Designing tasks so that students can work cooperatively (Paulus, 2005), dividing tasks between them for at least some of the project, is helpful for scheduling. It also enables a more individual marking scheme to be used, which in turn may encourage participation and reduce negative feelings towards the project.

An important aspect of designing online group projects is selecting collaboration tools. This review identified that discussion forums are still a common choice, often supported by other channels, including synchronous means of communicating. With the sudden move to online learning in 2020–2021 due to the Covid-19 pandemic (Rapanta et al., 2020) and increasing use of videoconferencing tools such as Zoom, Google hangouts and Microsoft Teams, the situation could change very rapidly.

**Fig. 3:**
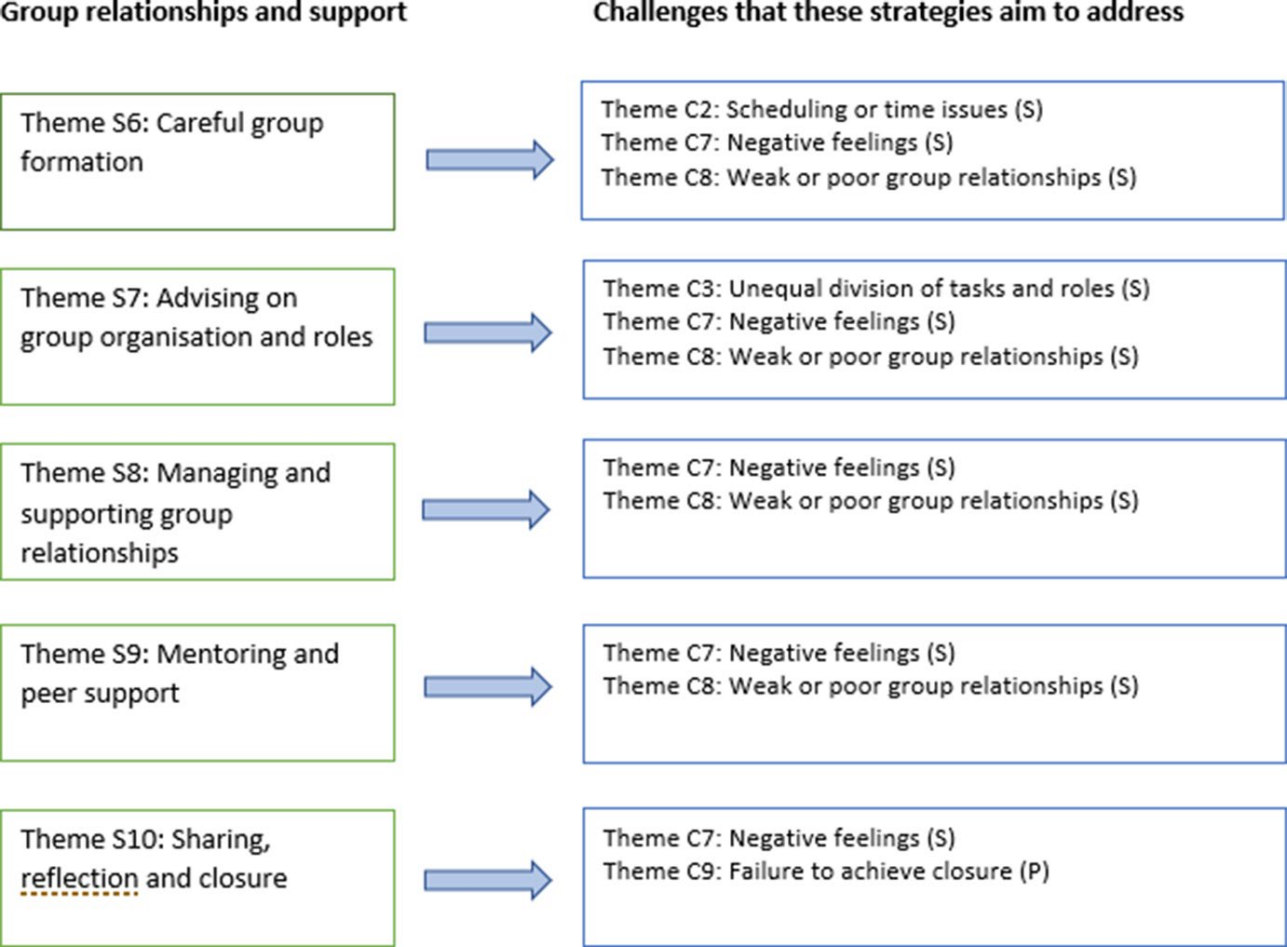
Strategies related to group relationships and support

We now move on to consider the second type of strategies identified (see Fig. 3) – ‘Group relationships and support’. These strategies are concerned with student support throughout the group work: ensuring groups keep on schedule, supporting them as they organise themselves, and helping them build and maintain strong working relationships. The strategies in this category are focused at the group level: group formation (S6), organisation (S7) and relationships (S8), peer support (S9) and reflection (S10).

As shown in Fig. 3, these strategies directly address the secondary ‘challenge’ themes (with the exception of C9) – particularly around counteracting negative feelings and poor relationships.

Returning to the Community of Inquiry framework, all three aspects of *social presence* (emotional expression; open communication; and group cohesion) need to be supported here. The ‘facilitating discourse’ element of *teaching presence* is particularly important for achieving this, as the tutor provides ongoing support for students’ online interactions. Although the tutor should not be overly present in online discussions, they should be available for both individual and group support, and able to provide encouragement and feedback at intervals, or intervene if problems arise.

### Linking challenges and strategies

The analysis presented in this paper identifies: links among challenges (Fig. 1); and links between strategies and challenges (Figs. 2 and 3). All the strategies contribute to addressing several challenges; and most of the challenges can be addressed by several strategies. For example, challenge C8 (weak or poor group relationships) is addressed by four strategies: S6 (group formation), S7 (group organisation), S8 (group relationships) and S9 (peer support). Addressing the challenge of weak or poor group relationships may therefore require actions related to all these strategy themes. Negative feelings (C7) are addressed, directly or indirectly, by all the strategies; this highlights the importance of considering all the strategies identified in this review. If an educator is aware of a particular challenge in their own context, the analysis presented in this paper should enable them to identify strategies to address it.

Finally, we offer comments about limitations of this review, and possible future work. During the reviewing process it became apparent that a number of the strategies identified, though proposed and implemented by educators, had not been formally evaluated. This means that, even though they are advocated and used by practitioners, the research evidence for their efficacy may not be present. We therefore propose such evaluation as necessary future research. This could be achieved, for example, by surveying/interviewing students and tutors about specific strategies used, and gaining their views on whether these were of value.

## Conclusion

In the context of a rapid global adoption of online learning, this paper has drawn together the experiences and findings of educators who have implemented online group projects. Based on a systematic literature review, the paper has thematically analysed the challenges posed by online group projects, and the strategies which have been proposed to address these challenges. The resulting nine ‘challenge’ themes and ten ‘strategy’ themes were presented and discussed, together with the relationships between them.

In summary, there is a need for educators to focus on two key strategic areas for addressing the challenges of online group projects:

1. Careful design of all aspects of the project, together with thorough initial guidance and preparation for students
2. Support for group relationships throughout the project.

We hope that this paper will help educators, particularly those new to online learning, to design and facilitate online group projects which students will find engaging, enjoyable and rewarding.

## Appendix

See Table

**Table 8:** The 61 papers identified for detailed analysis

Initial listing number	Title	First author	year	Score #1	score #2	Overall score
1	Student Perceptions of Social Task Development in Online Group Project Work	Morgan	2009	1	1	2
2	Creating and Collaborating: Students' and Tutors' Perceptions of an Online Group Project	Donelan	2018	1	1	2
3	Being Mindful May Not Make You a Team Player: Does Meditation Help or Hurt Online Group Work?	Tan	2017	2	2	4
6	Online Collaborative Learning Activities: The Perceptions of Culturally Diverse Graduate Students	Kumi-Yeboah	2017	1	2	3
9	Engaged Learning through Online Collaborative Public Relations Projects across Universities	Smallwood	2017	2	2	4
12	Features of Successful Group Work in Online and Physical Courses	Rezaei	2017	1	2	3
15	Active Knowledge Sharing in Online Group Work	Chang	2018	1	2	3
17	A Quantitative and Qualitative Evaluation of Student Participants' Contribution to Carrying out an OnlineInternational Collaborative Project on Education	Suzuki	2014	2	2	4
20	Student Perceptions of an Assessed, Online, Collaborative Activity	Haresnape	2015	2	2	4
21	Effective Use of Group Projects in Online Learning	Ekblaw	2016	1	1	2
22	Making Student Online Teams Work	Olson	2017	1	3	4
24	Analyzing the Social Knowledge Construction and Online Searching Behavior of High School Learners during a Collaborative Problem Solving Learning Activity: A Multi-Dimensional Behavioral Pattern Analysis	Lin	2016	1	3	4
27	Dimensions of Problem Based Learning: Dialogue and Online Collaboration in Projects	Andreasen	2013	2	2	4
28	Culture, Role and Group Work: A Social Network Analysis Perspective on an Online Collaborative Course	Stepanyan	2014	2	2	4
29	Collaborative Learning in an Online Course: A Comparison of Communication Patterns in Small and Whole Group Activities	Jahng	2010	2	2	4
31	Applying an Activity System to Online Collaborative Group Work Analysis	Choi	2010	1	1	2
32	Students Working Online for Group Projects: A Test of an Extended Theory of Planned Behaviour Model	Cheng	2017	2	2	4
34	Using Wikis for Online Group Projects: Student and Tutor Perspectives	Kear	2014	1	1	2
35	The Interrelationship of Emotion and Cognition when Students Undertake Collaborative Group Work Online: An Interdisciplinary Approach	Robinson	2013	1	1	2
36	Social Presence and Online Collaborative Small Group Work: A Socioconstructivist Account	Remesal	2013	1	1	2
37	Student-Centered Collaborative Learning in the Online Classroom: Perceptions of Virtual Group Projects	Mandernach	2007	1	1	2
38*	Does a Case-Based Online Group Project Increase Students' Satisfaction with Interaction in Online Courses?	Lee	2016	1	3	4
39	Assessment of Individual Student Performance in Online Team Projects	Alden	2011	1	1	2
40	How Much "Group" Is There in Online Group Work?	Lowes	2014	1	2	3
45	Online Group Work Design: Processes, Complexities, and Intricacies	Kleinsasser	2016	1	2	3
46	An Interdisciplinary Team Project: Psychology and Computer Science Students Create Online Cognitive Tasks	Flannery	2014	1	1	2
47	Promoting Collaboration in a Project-Based E-Learning Context	Papanikolaou	2010	2	2	4
49	Key Components of Online Group Projects: Faculty Perceptions	Wade	2016	1	2	3
50	Online Group Work Patterns: How to Promote a Successful Collaboration	Oliveira	2011	1	1	2
53	Challenges Facing Group Work Online	Chang	2016	1	1	2
55*	Technologies That Assist in Online Group Work: A Comparison of Synchronous and Asynchronous Computer Mediated Communication Technologies on Students' Learning and Community	Rockinson-Szapkiw	2015	2	2	4
59	Teacher Perspectives on Online Collaborative Learning: Factors Perceived as Facilitating and Impeding Successful Online Group Work	An	2008	1	2	3
61	A Performance Evaluation of the Collaborative Efforts in an Online Group Research Project	Brown	2008	1	2	3
63	Enhancing Project-Based Learning through Online Between-Group Collaboration	Lou	2004	2	1	3
64	Learning to Solve Complex Problems through Between-Group Collaboration in Project-Based Online Courses	Lou	2004	2	1	3
65	Faculty Perceptions of Online Group Work	Morgan	2014	1	1	2
66	Group Projects: Student Perceptions of the Relationship between Social Tasks and a Sense of Community in Online Group Work	Cameron	2009	1	1	2
68	Analyzing the Social Knowledge Construction Behavioral Patterns of an Online Synchronous Collaborative Discussion Instructional Activity Using an Instant Messaging Tool: A Case Study	Hou	2011	2	2	4
74	Online Collaborative Learning in a Project-Based Learning Environment in Taiwan: A Case Study on Undergraduate Students' Perspectives	Zhang	2009	1	2	3
76	Online Graduate Study Health Care Learners' Perceptions of Group Work and Helpful Instructional Behaviors	Bergeron	2006	2	2	4
77	Are Interpersonal Relationships Necessary for Developing Trust in Online Group Projects?	Wade	2011	1	1	2
78	Designing and Fostering Effective Online Group Projects	Scherling	2011	1	1	2
79	How Do Students Define Their Roles and Responsibilities in Online Learning Group Projects?	Williams	2011	1	2	3
81	Asynchronous Online Collaboration as a Flexible Learning Activity and an Authentic Assessment Method in an Undergraduate Mathematics Course	Mallet	2008	1	3	4
83	Investigating the Development of Work-Oriented Groups in an e-Learning Environment	Yu	2012	1	1	2
84	Overcoming Student Resistance to Group Work: Online Versus Face-to-Face	Smith	2011	1	1	2
85	A Collaborative Online Project between New Zealand and New York	Zhu	2005	2	2	4
86	Online Project-Based Learning: How Collaborative Strategies and Problem Solving Processes Impact Performance	Thomas	2005	1	1	2
91*	The Benefits and Limitations of Online Group Work in a Teacher Education Program	An	2009	1	2	3
94	Instructor Immediacy Strategies to Facilitate Group Work in Online Graduate Study	Melrose	2007	2	2	4
95	The Relationship between Adopting a Synchronous Medium and Participation in Online Group Work: An Explorative Study	Hrastinski	2006	2	2	4
97	Quality Issues of Group Work and Leadership Emergence in E-Learning: Case Study	Fisher	2005	1	1	2
99*	Online Collaboration: Making It Work	Hathorn	2002	1	2	3
*From first catch-up run:*						
102	Evaluating the Impact of Online Discussion Boards on Student Engagement with Group Work	Delaney	2019	1	3	4
*From second catch-up run*						
105	Vertical versus Shared E-Leadership Approach in Online Project-Based Learning: A Comparison of Self-Regulated Learning Skills, Motivation and Group Collaboration Processes	Yilmaz	2020	1	1	2
107	Students' Perceptions of Challenges and Solutions to Face-to-Face and Online Group Work	Bakir	2020	1	1	2
108	Online Group Work with a Large Cohort: Challenges and New Benefits	Hurst	2020	1	1	2
109	Favorability of Strategies to Facilitate Online Group Work	Kritzer	2020	2	2	4
110*	Integration of Examination Strategies in E-Learning Platform for Assessment of Collaborative Activities	Aouine	2020	2	2	4
111	Impact of Intercultural Online Collaboration Project for Pre-Service Teachers	Hur	2020	2	2	4
113	A Workflow-Based Solution to Support the Assessment of Collaborative Activities in E-Learning: A Design Founded on IMS-LD Meta-Model	Aouine	2019	1	2	3

^***^*Unable to access paper therefore not included in analysis*

8

## References

[CR1] Alden J (2011). Assessment of individual student performance in online team projects. Online Learning..

[CR2] An H, Kim S (2009). The benefits and limitations of online group work in a teacher education program. Journal of Technology and Teacher Education.

[CR3] An H, Kim S, Kim B (2008). Teacher perspectives on online collaborative learning: factors perceived as facilitating and impeding successful online group work. Contemporary Issues in Technology and Teacher Education..

[CR4] Andreasen LB, Nielsen JL (2013). Dimensions of problem based learning: Dialogue and online collaboration in projects. Journal of Problem Based Learning in Higher Education.

[CR5] Aouine A, Mahdaoui L (2020). Integration of examination strategies in e-learning platform for assessment of collaborative activities. International Journal of Information and Communication Technology Education.

[CR6] Aouine A, Mahdaoui L, Moccozet L (2019). A workflow-based solution to support the assessment of collaborative activities in e-learning. The International Journal of Information and Learning Technology.

[CR7] Bakir N, Humpherys S, Dana K (2020). Students’ perceptions of challenges and solutions to face-to-face and online group work. Information Systems Education Journal.

[CR8] Bakken, R. (2018). Challenges to managing virtual teams and how to overcome them. Harvard division of continuing education. Blog post available at https://www.extension.harvard.edu/professional-development/blog/challenges-managing-virtual-teams-and-how-overcome-them Accessed 23/7/2020.

[CR9] Bergeron K, Melrose S (2006). Online graduate study health care learners’ perceptions of group work and helpful instructional behaviors. Journal of Educational Technology.

[CR10] Brindley JE, Walti C, Blaschke LM (2009). Creating effective collaborative learning groups in an online environment. The International Review of Research in Open and Distributed Learning.

[CR11] Brown LA, Eastham NP, Ku H-Y (2008). A performance evaluation of the collaborative efforts in an online group research project. Performance Improvement Quarterly.

[CR12] Cameron BA, Morgan K, Williams KC, Kostelecky KL (2009). Group projects: student perceptions of the relationship between social tasks and a sense of community in online group work. American Journal of Distance Education.

[CR13] Chandler K (2022). Adapting voice-centred relational method to understand students’ experiences of synchronous online tuition. International Journal of Research & Method in Education.

[CR14] Chang B (2018). Active knowledge sharing in online group work. New Horizons in Adult Education & Human Resource Development.

[CR15] Chang B, Kang H (2016). Challenges facing group work online. Distance Education.

[CR16] Cheng EWL (2017). Students working online for group projects: A test of an extended theory of planned behaviour model. Educational Psychology.

[CR17] Choi H, Kang M (2010). Applying an activity system to online collaborative group work analysis: Online group work analysis. British Journal of Educational Technology.

[CR18] Delaney D, Kummer T-F, Singh K (2019). Evaluating the impact of online discussion boards on student engagement with group work: Evaluating impact of online discussion boards. British Journal of Educational Technology.

[CR19] Donelan H, Kear K (2018). Creating and collaborating: students’ and tutors’ perceptions of an online group project. The International Review of Research in Open and Distributed Learning.

[CR20] Ekblaw R (2016). Effective use of group projects in online learning. Contemporary Issues in Education Research..

[CR21] Fiock H (2020). Designing a community of inquiry in online courses. The International Review of Research in Open and Distributed Learning.

[CR22] Fisher M, Tucker D, Silverberg D (2005). Quality issues of group work and leadership emergence in e-learning: Case study. Journal of Educational Technology Systems.

[CR23] Flannery KA, Malita M (2014). An Interdisciplinary team project: psychology and computer science students create online cognitive tasks. College Teaching.

[CR24] Garrison DR, Anderson T, Archer W (2000). Critical Inquiry in a Text-Based Environment: Computer Conferencing in Higher Education. The Internet and Higher Education.

[CR25] Gunawardena C, Zittle F (1997). Social presence as a predictor of satisfaction within a computer-mediated conferencing environment. American Journal of Distance Education.

[CR26] Gusenbauer M, Haddaway NR (2019). Which academic search systems are suitable for systematic reviews or meta-analyses? Evaluating retrieval qualities of Google Scholar, PubMed, and 26 other resources. Research Synthesis methods.

[CR27] Haresnape J (2015). Student perceptions of an assessed, online, collaborative activity. Practitioner Research in Higher Education.

[CR28] Hathorn LG, Ingram AL (2002). Online collaboration: Making It Work. Educational Technology.

[CR29] Herrington J, Reeves TC, Oliver R (2010). A guide to authentic elearning.

[CR30] Hiltz SR, Goldman R (2005). (2005) Learning Together Online: Research on Asynchronous Learning Networks.

[CR31] Hou H-T, Wu S-Y (2011). Analyzing the social knowledge construction behavioral patterns of an online synchronous collaborative discussion instructional activity using an instant messaging tool: A case study. Computers & Education.

[CR32] Hrastinski S (2006). The relationship between adopting a synchronous medium and participation in online group work: An explorative study. Interactive Learning Environments.

[CR33] Hur JW, Shen YW, Cho M-H (2020). Impact of intercultural online collaboration project for pre-service teachers. Technology, Pedagogy and Education.

[CR34] Hurst GA (2020). Online group work with a large cohort: challenges and new benefits. Journal of Chemical Education.

[CR35] Jahng N, Nielsen WS, Chan EKH (2010). Collaborative learning in an online course: a comparison of communication patterns in small and whole group activities. Journal of Distance Education.

[CR36] Ke F (2010). Examining online teaching, cognitive, and social presence for adult students. Computers & Education.

[CR37] Kear K, Donelan H, Williams J (2014). Using wikis for online group projects: Student and tutor perspectives. The International Review of Research in Open and Distributed Learning.

[CR38] Kleinsasser R, Hong Y-C (2016). Online group work design: processes, complexities, and intricacies. TechTrends.

[CR39] Kreijns K, Kirschner PA, Jochems W (2003). Identifying the pitfalls for social interaction in computer-supported collaborative learning environments: A review of the research. Computers in Human Behavior.

[CR40] Kritzer J, Bogan J (2020). Favorability of strategies to facilitate online group work. Journal of Educators Online.

[CR41] Kumi-Yeboah A, Yuan G, Dogbey J (2017). Online collaborative learning activities: The perceptions of culturally diverse graduate students. Online Learning Journal.

[CR42] Lee SJ, Ngampornchai A, Trail-Constant T, Abril A, Srinivasan S (2016). Does a case-based online group project increase students' satisfaction with interaction in online courses?. Active Learning in Higher Education.

[CR43] Lin C-L, Hou H-T, Tsai C-C (2016). Analyzing the social knowledge construction and online searching behavior of high school learners during a collaborative problem solving learning activity: A multi-dimensional behavioral pattern analysis. The Asia-Pacific Education Researcher.

[CR44] Lombardi, M. M. (2007). Authentic learning for the 21st century: An overview. Educause LearningInitiative. Retrieved from: https://library.educause.edu/resources/2007/1/authentic-learning-for-the-21st-century-an-overview

[CR45] Lou Y (2004). Learning to solve complex problems through between-group collaboration in project-based online courses. Distance Education.

[CR46] Lou Y, Kim MacGregor S (2004). Enhancing project-based learning through online between-group collaboration. Educational Research and Evaluation.

[CR47] Lowes, S. (2014.). How much ‘group’ is there in online group work. Journal of Asynchronous Learning Networks. 18(1)

[CR48] Mallet DG (2008). Asynchronous online collaboration as a flexible learning activity and an authentic assessment method in an undergraduate mathematics course. EURASIA Journal of Mathematics, Science and Technology Education.

[CR49] Mandernach BJ, Donnelli E, Dailey A (2007). Student-centered collaborative learning in the online classroom: perceptions of virtual group projects. Journal on School Educational Technology.

[CR50] McConnell D (2005). Examining the dynamics of networked e-learning groups and communities. Studies in Higher Education.

[CR51] McConnell D (2006). E-learning Groups and Communities.

[CR52] Melrose S, Bergeron K (2007). Instructor immediacy strategies to facilitate group work in online graduate study. Australasian Journal of Educational Technology.

[CR53] Morgan K, Cameron BA, Williams KC (2009). Student perceptions of social task development in online group project work. The Quarterly Review of Distance Education.

[CR54] Morgan K, Williams KC, Cameron BA, Wade CE (2014). Faculty perceptions of online group work. The Quarterly Review of Distance Education.

[CR55] Newman M, Gough D, Zawacki-Richter O, Kerres M, Bedenlier S, Bond M, Buntins K (2020). Systematic reviews in educational research: methodology, perspectives and application. Systematic reviews in educational research.

[CR56] Oliveira I, Tinoca L, Pereira A (2011). Online group work patterns: How to promote a successful collaboration. Computers & Education.

[CR57] Oliver R, Herrington A, Herrington J, Reeves TC (2007). Representing authentic learning designs supporting the development of online communities of learners. Journal of Learning Design.

[CR58] Olson J, Kalinski R (2017). Making student online teams work. The Quarterly Review of Distance Education.

[CR59] Papanikolaou K, Boubouka M (2010). Promoting collaboration in a project-based e-learning context. Journal of Research on Technology in Education.

[CR60] Paulus TM (2005). Collaborative and cooperative approaches to online group work: The impact of task type. Distance Education.

[CR61] Payne BK, Monk-Turner E (2006). Students' perceptions of group projects: The role of race, age, and slacking. College Student Journal.

[CR62] Rapanta C, Botturi L, Goodyear P, Guàrdia L, Koole M (2020). Online university teaching during and after the Covid-19 crisis: refocusing teacher presence and learning activity. Postdigital Science and Education.

[CR63] Remesal A, Colomina R (2013). Social presence and online collaborative small group work: A socioconstructivist account. Computers & Education.

[CR64] Rezaei A (2017). Features of successful group work in online and physical courses. The Journal of Effective Teaching.

[CR65] Roberts T, McInnerney J (2007). Seven problems of online group learning (and Their Solutions). Educational Technology and Society.

[CR66] Robinson K (2013). The interrelationship of emotion and cognition when students undertake collaborative group work online: An interdisciplinary approach. Computers & Education.

[CR67] Rockinson-Szapkiw A, Wendt J (2015). Technologies that assist in online group work: A comparison of synchronous and asynchronous computer mediated communication technologies on students' learning and community. Journal of Educational Multimedia and Hypermedia..

[CR68] Scherling SE (2011). Designing and fostering effective online group projects. Adult Learning.

[CR69] Shiue Y, Chiu C, Chang C (2010). Exploring and mitigating social loafing in online communities. Computers in Human Behavior.

[CR70] Smallwood AMK, Brunner BR (2017). Engaged learning through online collaborative public relations projects across universities. Journalism & Mass Communication Educator.

[CR71] Smith GG, Sorensen C, Gump A, Heindel AJ, Caris M, Martinez CD (2011). Overcoming student resistance to group work: Online versus face-to-face. The Internet and Higher Education.

[CR72] Stepanyan K, Mather R, Dalrymple R (2014). Culture, role and group work: A social network analysis perspective on an online collaborative course: SNA perspective on an online collaborative course. British Journal of Educational Technology.

[CR73] Suzuki, C., Ishida, K., Yoshihara, S., Schultheis, K., & Riedhammer, B. (2014). A quantitative and qualitative evaluation of student participants’ contribution to carrying out an online international collaborative project on education. *Proceedings of the 2014 EUROCALL Conference*, Groningen, The Netherlands, 345–351. 10.14705/rpnet.2014.000243

[CR74] Tan Y, Molinari C (2017). Being mindful may not make you a team player: does meditation help or hurt online group work?. Journal of Educators Online.

[CR75] Thomas WR, MacGregor SK (2005). Online project-based learning: how collaborative strategies and problem solving processes impact performance. Journal of Interactive Learning Research.

[CR76] Tuckman BW (1965). Developmental sequence in small groups. Psychological Bulletin.

[CR77] Tuckman BW, Jensen MC (1977). Stages of small group development revisited. Group and Organizational Studies.

[CR78] Wade CE, Cameron BA, Morgan K (2016). Key components of online group projects. The Quarterly Review of Distance Education.

[CR79] Wade CE, Cameron BA, Morgan K, Williams KC (2011). Are interpersonal relationships necessary for developing trust in online group projects?. Distance Education.

[CR80] Williams KC, Morgan K, Cameron BA (2011). How do students define their roles and responsibilities in online learning group projects?. Distance Education.

[CR81] Winterbotham et al (2018) Department for Education Employer Skills Survey Report 2017. https://assets.publishing.service.gov.uk/government/uploads/system/uploads/attachment_data/file/746493/ESS_2017_UK_Report_Controlled_v06.00.pdf

[CR82] Wong B, Chiu Y, Copsey-Blake M, Nikolopoulou M (2022). A mapping of graduate attributes: What can we expect from UK university students?. Higher Education Research & Development.

[CR83] Yilmaz R, Yilmaz GK (2020). Vertical versus shared e-leadership approach in online project-based learning: A comparison of self-regulated learning skills, motivation and group collaboration processes. Journal of Computing in Higher Education.

[CR84] Yu C-P, Kuo F-Y (2012). Investigating the development of work-oriented groups in an e-learning environment. Educational Technology & Society.

[CR85] Yu Z, Li M (2022). A bibliometric analysis of community of Inquiry in online learning contexts over twenty-five years. Education and Information Technologies.

[CR86] Zhang K, Peng SW, Hung J (2009). Online collaborative learning in a project-based learning environment in Taiwan: A case study on undergraduate students’ perspectives. Educational Media International.

[CR87] Zhu Y, Gareis E, Bazzoni JO, Rolland D (2005). A collaborative online project between New Zealand and New York. Business Communication Quarterly.

